# *Trichinella spiralis*, potential model nematode for epigenetics and its implication in metazoan parasitism

**DOI:** 10.3389/fphys.2013.00410

**Published:** 2014-01-10

**Authors:** Fei Gao, Rui Wang, Mingyuan Liu

**Affiliations:** ^1^Science and Technology Department, BGI-Shenzhen, Beishan Industrial ZoneShenzhen, China; ^2^Key Lab for Zoonosis Research, Ministry of Education, Institute of Zoonosis, Jilin UniversityChangchun, China; ^3^Jiangsu Co-innovation Center for Prevention and Control of Important Animal Infectious Diseases and ZoonosesYangzhou, China

**Keywords:** *trichinella spiralis*, epigenetics, model nematode, metazoan, parasitism

## Abstract

The recent discovery of DNA methylation in the nematode *T.spiralis* may raise the possibility of using it as a potential model organism for epigenetic studies instead of *C. elegans*, which is deficient in this important epigenetic modification. In contrast to the free-living nematode *C. elegans, T. spiralis* is a parasitic worm that possesses a complicated life cycle and undergoes a complex developmental regulation of genes. We emphasize that the differential methylomes in the different life-history stages of *T. spiralis* can provide insight on how DNA methylation is triggered and regulated. In particular, we have demonstrated that DNA methylation is involved in the regulation of its parasitism-related genes. Further computational analyses indicated that the regulatory machinery for DNA methylation can also be found in the *T. spiralis* genome. By a logical extension of this point, we speculate that comprehensively addressing the epigenetic machinery of *T. spiralis* may help to understand epigenetics in invertebrates. Furthermore, considering the implication of epigenetics in metazoan parasitism, using *T. spiralis* as an epigenetic model organism may further contribute to drug development against metazoan parasites.

## Current model organisms in epigenetic studies

Research activities using model organisms have provided critical breakthroughs in understanding fundamental questions in biology. The most popular animal models are the nematode *Caenorhabditis elegans*, the fruit fly *Drosophila melanogaster*, the zebrafish *Danio rerio*, the African clawed frog *Xenopus laevis*, the chicken *Gallus gallus domesticus*, the mouse *Mus musculus* and the rat *Rattus norvegicus* (Cogburn et al., 2007; Jenner and Wills, 2007; Kennedy, 2008). Benefiting from technological advancements, the complete genomes of multiple model organisms have been obtained. However, our knowledge about how the information encoded in their genomes is regulated or interpreted is still limited. Thus, understanding epigenetic phenomena has become a major focus of research activity in the current post-genomics era.

The term epigenetics refers to the study of any potentially stable and ideally heritable change that alters gene expression without altering DNA sequences in an organism (Goldberg et al., 2007). Among a variety of mechanisms involved, DNA methylation and post-translationally modified histones within their N termini can result in changes in the accessibility of chromatin to chromatin-remodeling complexes, transcriptional complexes and polymerases. Epigenetic studies also necessarily require good experimental models. Indeed, various model organisms have been applied to date, through which a wealth of knowledge has been acquired from several landmark epigenetic discoveries (David Allis and Danny Reinberg, 2007). Different model organisms offer different advantages, and all are important for learning about the processes and mechanisms involved in epigenetic regulation. For example, multiple species of yeast have been used as model systems to study chromatin structure (David Allis and Danny Reinberg, 2007). Because DNA methylation is not observed in yeast, the presence of DNA methylation in the fungus *Neurospora crassa* made it a model organism for DNA methylation studies and contributed to the discovery of repeat-induced point mutation (RIP), which is a genome defense mechanism (David Allis and Danny Reinberg, 2007). Histone variants, the first histone acetyltransferase, histone lysine methylation, histone phosphorylation and one RNAi pathway were discovered in the protozoan *Tetrahymena thermophila* (David Allis and Danny Reinberg, 2007). The studies on the fruit fly *D. melanogaster* have led to the discovery of chromatin remodeling proteins and histone modifying proteins (Lecuyer et al., 2007). Plants, such as *Arabidopsis thaliana*, have epigenetic mechanisms as sophisticated as those of mammals, including RNAi pathways, DNA methylation, histone modification, and chromosome remodeling complexes (Henderson and Jacobsen, 2007). Finally, mice, as mammals, are more similar to humans than any of these model systems and are used as models for epigenetic research, particularly in embryology, stem cell research and environmental studies, including the effects of behavior and nutrition on epigenetic states.

## Biology and genome of *T. spiralis*

**Figure 1:**
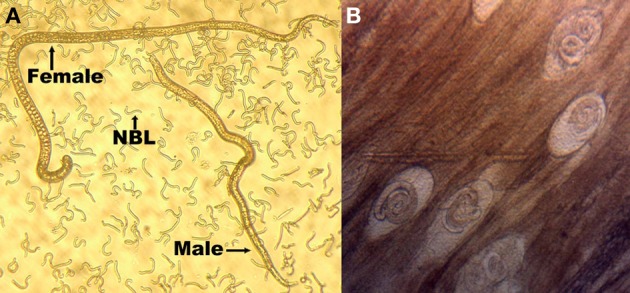
**Three main life-stage forms of *T. spiralis*. (A)** Adults and new born larvae (NBL) and **(B)** infective muscle larvae. Female adults, which develop in the small intestine, will produce new-born larvae, which then disseminate through the bloodstream, invade skeletal muscles, and encyst in a collagen capsule to form a new generation of muscle larvae.

In contrast to the free-living nematode *C. elegans, Trichinella spiralis* (*T. spiralis*) is a parasitic worm that possesses a complicated life cycle and undergoes complex developmental regulation of its genes (Figure 1) (Mitreva and Jasmer, 2006). *T. spiralis* begins its life cycle as muscle larvae contained in infected meat. With the aid of host gastric juices, the muscle larvae are then released into the host's stomach and mature into sexually active adults in the host's intestines. After the second generation larvae are born, they migrate throughout the entire body of the host and invade the skeletal muscles. The second generation larvae can survive in host for years (up to 40 years in humans), and this hypobiotic stage is maintained until being ingested by a new host, thereby the new generation begin (Mitreva and Jasmer, 2006).

*T. spiralis* exhibits a wide, global distribution, and this species is the most important etiological agent to cause disease in humans (Mitreva and Jasmer, 2006). Trichinellosis affects approximately ten million people globally (Tantrawatpan et al., 2013), with the common signs and symptoms in humans including fever, diarrhea, periorbital oedema and myalgia. Humans acquire this disease by ingesting raw or insufficiently cooked meat containing the infective larvae of *T. spiralis* (Wang et al., 2013). This disease not only is a public-health hazard but also represents an economic problem in porcine animal production and food safety (Mitreva and Jasmer, 2010). However, until now the mechanism by which *T. spiralis* infective larvae distinguish, attack, creep within the intestinal epithelium, and create their intramuscular niches have been inadequately explained, which has made the therapy against trichinellosis much more difficult (Basyoni and El-Sabaa, 2013).

In the evolution of the Nematoda, *T. spiralis* is a member of a clade that diverged early. It differs substantially in its biological and molecular characteristics from other crown groups. The completion of the *T. spiralis* draft genome enables further investigations on this interesting parasitic nematode, particularly with respect to either its evolutional position or comparative and functional genomics (Mitreva et al., 2011).

## Preliminary discovery in epigenetics of *T. spiralis*

Extensive epigenetic studies have been performed on the genome of the well-annotated model nematode *C. elegans*, addressing its functional genomic elements including histone modifications (Gerstein et al., 2010; Liu et al., 2011). However, the most studied epigenetic mechanism, DNA methylation, is absent in *C. elegans* (Simpson et al., 1986). By extension, conventional thinking has held that DNA methylation is missing throughout the nematode phylum. However, recent progress has been made with the discovery, for the first time, of the presence of DNA methylation in *T. spiralis* (Gao et al., 2012). Not only have genes that are homologous to canonical dnmt1 and dnmt3 in the *T. spiralis* genome been identified, but also the presence of DNA methylation was probed directly by several methods. As *T. spiralis* belongs to a basal clade of Nematoda, having diverged from *C. elegans* several hundred million years ago in the late Precambrian (Mitreva et al., 2011), this species likely retained DNA methylation machinery that may have been present in the ancestral nematode. Furthermore, potential homologs of canonical histone-modifying enzymes, chromatin remodeling factors and other relevant proteins were also revealed in the released draft genome sequence (Mitreva et al., 2011) and in our own computational analyses (data not shown), suggesting the presence of a fully developed epigenetic system in *T. spiralis*. These results revealed a new and unique characterization of this species that differs from other nematode species.

## *T. spiralis* may provide a new system for probing epigenetic regulation

Model organisms not only have some characteristics in common, such as easy culture, non-specialist living requirements and amenability in experimental manipulation but also have one or more special features that qualify them to be outstanding subjects in research. For example, in *C. elegans*, the fate of each constituent cell can be exactly traced, and in *D. melanogaster*, mutations are easily induced with the resulting phenotypes being easily observed.

*T. spiralis* is also an easy organism to raise in the lab, with high fertility, relatively short generation time and easy accessibility of all life-history stages, which ensure that it is an accessible model to study. More importantly, it has special features that are advantageous to being an epigenetics model organism compared with other existing non-mammalian model organisms. First, as a nematode, *T. spiralis* appears to have developed a relatively complete epigenetic system. In addition to evolutionally conserved histone modifications, similar characteristics of the *T. spiralis* methylome compared with vertebrate genomes were found, although methylated cytosines are relatively sparse. The methylation of transposable elements was previously viewed as a unique feature of vertebrate genomes and was thought to be mostly absent from invertebrates (Suzuki and Bird, 2008). However, in the *T. spiralis* genome, transposable elements appear to be more heavily methylated than the genomic background. Furthermore, the DNA methylation levels of gene upstream regions have a negative correlation with gene expression levels, and non-expressed genes in particular had different patterns of DNA methylation with the methylation levels in their upstream regulatory regions being higher than in the coding sequences. This phenomenon fits with the model of vertebrate animals but is different from some non-vertebrate organisms, such as silkworm (Xiang et al., 2010). Second, by screening and sequencing methylated DNA present at the new-born, larval, and adult stages, virtually no methylation during the new-born stage was found. In the larval stage, when the worms move from the intestine to the muscle, however, methylation begins to occur, with adult worms carrying the most methylated DNA. The methylation machinery appears to be suddenly switched to the “on” state in the muscle larvae stage from the “off” state in the NBL stage. This finding is in stark contrast to DNA methylation patterns in other animals, including humans, which generally stay consistent throughout the organism's lifetime. Thus, *T. spiralis* may provide an excellent model for studying the pathways leading to the initiation, establishment, maintenance and plasticity of methylation states. Understanding such pathways will be extremely useful in dissecting the functions of DNA methylation in organismal development or differentiation and in disease occurrence.

## *T. spiralis* may provide a new system for studying the epigenetic mechanism of metazoan parasitism

Parasites, either protozoan or metazoan, must be able to rapidly transit between complex life cycles and use long-term antigenic variation to avoid immune attacks in their hosts. To achieve this outcome, the parasite must initiate complex differentiation in response to environmental cues, which requires drastic and rapid alterations of their gene expression profiles. The transcriptional regulation of gene expression is therefore employed.

As an essential means of transcriptional regulation, epigenetics has emerged as a crucial aspect of parasite biology over the past decade. A major focus of investigation has been the characterization of histone modifications and the identification of the responsible enzymes. Currently, intensive studies in the protozoan parasites *Plasmodium falciparum, Toxoplasma gondii* and *Trypanosoma brucei* have been performed, which was reviewed nicely by Croken et al. (2012). The existence and functional roles of histone modifications in the epigenetic control of platyhelminth schistosome transcription have also been established [reviewed in Geyer and Hoffmann (2012)]. Through these studies, the relative conservation of histone-modifying enzymes across different species, including both protozoan and metazoan parasites, has been associated with evolutionarily conserved functions.

Observations of the most highly studied epigenetic mechanism of DNA methylation in mammals appeared variable in parasites. Among the protozoan parasites, *T. brucei* and *Entamoeba histolytica* have a DNA methyltransferase and methylated DNA detectable by mass spectrometry (Fisher et al., 2004; Militello et al., 2008). The genomes of *T. gondii* and Plasmodium and Cryptosporidium species do encode candidate methyltransferases, although no detectable methylated cytosines were discovered in previous studies (Gissot et al., 2008). The detection of DNA methylation is highly dependent on the sensitivity of the applied technology, and thus DNA methylation may be restricted to a small number of loci that were not detected by the methodology used. In the metazoan parasites, a previous study convincingly demonstrated that the platyhelminth *Schistosoma mansoni* contained a methylated genome and that methylation was found to be differentially present across the parasite's lifecycle (Geyer et al., 2011). For other nematodes in addition to *C. elegans* and *T. spiralis*, although a canonical DNA methyltransferase (dnmt3) homolog among 11 nematodes has not been found, we should not exclude the possibility of the existence of cytosine methylation in their genomes. Exceptionally, the silkworm uses DNA methylation, but its genome does not contain dnmt3 (Xiang et al., 2010). Moreover, the model protozoan ciliate does in fact utilize DNA methylation during a certain part of its life cycle (Bracht et al., 2012) even though no homologs of dnmts were found in its genome. Taken together, DNA methylation might also be an ancient epigenetic mechanism occurring in parasites and may play essential roles in gene regulation. Recent innovations in high-throughput sequencing may enable researchers to infer methylation at a single-base resolution [10], thereby raising the possibility of a thorough investigation of the conservation and divergence of methylation patterns in different parasites. As a parasitic nematode, *T. spiralis* shares many characteristics with other parasites regarding parasitism. Parasitism-related genes were also found to be regulated by DNA methylation between the life cycle stages of *T. spiralis*, suggesting the potential biological implications of epigenetics in parasitism. Although further studies are required to reveal the implication of other epigenetic mechanisms; based on its fully developed epigenetic system, *T. spiralis* may serve as a good model for studying the implication of epigenetics in metazoan parasitism.

**Figure 2:**
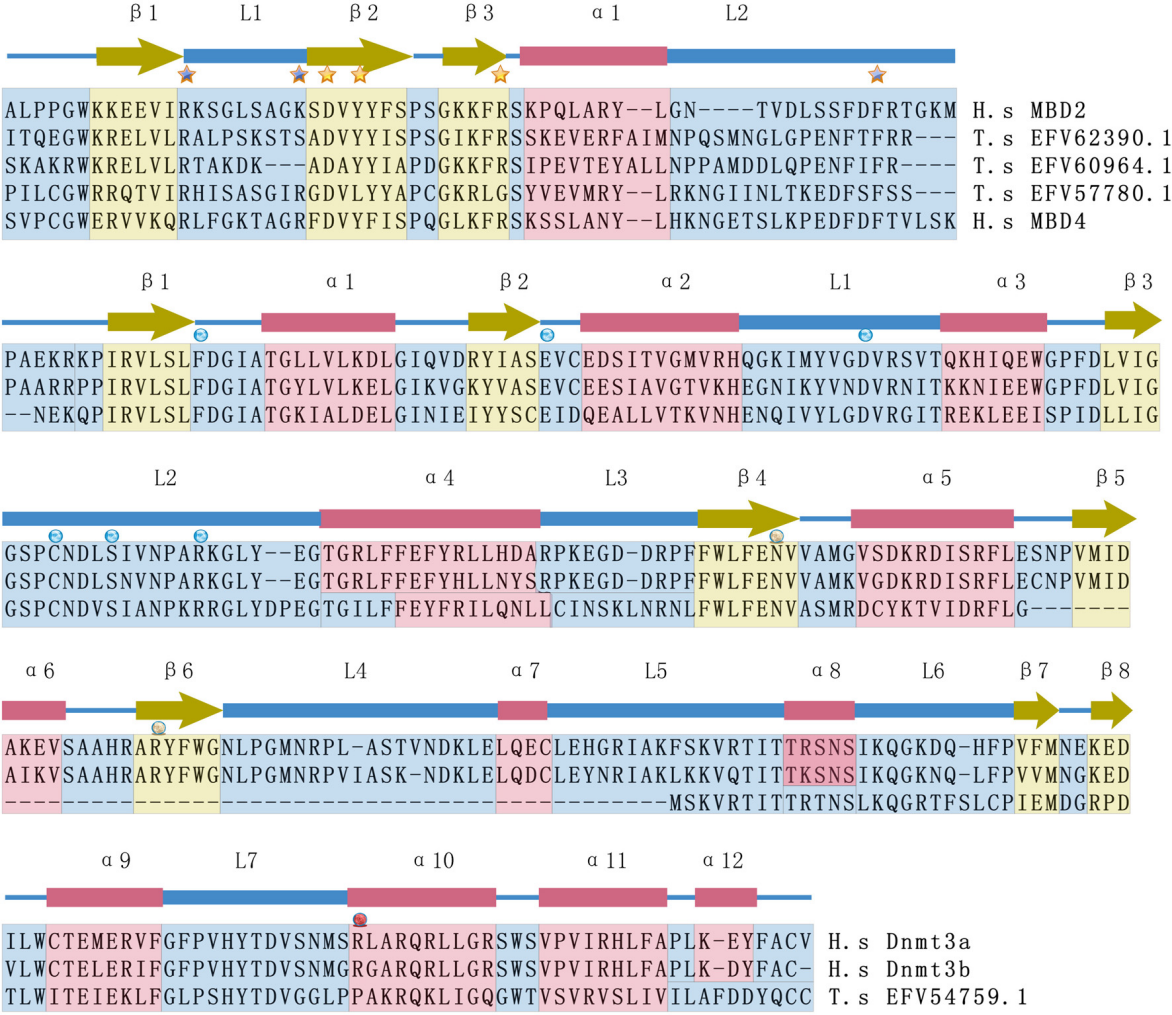
**Comparison of the secondary structures of MBD domains and Dnmt3 catalytic domains between *T. spiralis* and *H. sapiens***. The secondary structures of human MBD2 and Dnmt3a are indicated as blocks, including alpha helices (α), beta sheets (β), and loops (L), that are marked with different colors (blue = alpha helices, yellow = beta sheets, and red = loops). Important residues responsible for methyl-CpG binding are marked with asterisks. The functional sites of the Dnmt3 catalytic domain are marked with circles, including reported S-Adenosyl methionine (SAM) binding sites and active sites. H.s and T.s are short for *H. sapiens* and *T. spiralis*, respectively. T.s EFV 62390.1, T.s EFV 60964.1 and T.s EFV57780 are proteins in *T. spiralis* that show high sequence similarity with human Mbd proteins. T.s EFV54759.1 is a protein of *T.spiralis* that has sequence similarity with human Dnmt3 protein.

More importantly, understanding epigenetic regulation using the *T. spiralis* model opens new methods for the diagnosis and treatment of metazoan parasites, especially with respect to the development of new drugs affecting the epigenetic machinery. To develop new compounds that interfere with pathogenic states, the targeted epigenetic regulators should be specific compared with their host orthologs. Previous analyses on class I HDACs (SmHDAC8) and sirtuins (SmSirt1 and 2) in metazoan schistosomes have shown such potential regarding schistosomes (Pierce et al., 2011). Our analyses on dnmts and mbd-like proteins of *T. spiralis* also show notable differences in their catalytic domains compared with their mammalian orthologs (Figure 2). Therefore, given the importance of epigenetics in metazoan parasite biology, drug discovery by targeting epigenetic regulators should be promising as a paradigm for drug development against metazoan parasites.

## Conclusion

We highlight that the current findings on the DNA methylation of *T. spiralis* may raise the possibility of using it as a potential model organism in epigenetic studies. We emphasize that the differential methylomes in the different life-history stages of *T. spiralis* can provide insight into how DNA methylation is triggered and regulated. In particular, *T. spiralis* is a parasitic worm that diverged early in the evolution of nematodes, and epigenetics is involved in regulation of its parasitism-related genes. By a logical extension of this point, we speculate that comprehensively addressing the epigenetic machinery of *T. spiralis* may help to deeply understand the implication of epigenetics in metazoan parasitism and further contribute to the drug development against metazoan parasites.

### Conflict of interest statement

The authors declare that the research was conducted in the absence of any commercial or financial relationships that could be construed as a potential conflict of interest.
